# Platelet-related hematologic markers and genetic associations of aspirin resistance in kawasaki disease

**DOI:** 10.3389/fmolb.2026.1807254

**Published:** 2026-04-01

**Authors:** Linjie Jiang, Xiaoting Ding, Wan Yang, Xilian Luo, Kaining Chen, Lanyan Fu, Yufen Xu, Huazhong Zhou, Xiaoxue Li, Caiting Xiao, Xiaoqiong Gu, Xiangna Yang, Chunjiao Wei, Zhouping Wang, Jianrui Wei, Lei Pi

**Affiliations:** 1 Department of Cardiac Center, Guangzhou Women and Children’s Medical Center, Guangzhou Medical University, Guangzhou, China; 2 Department of Clinical Biological Resource Bank, Guangzhou Women and Children’s Medical Center, Guangzhou Medical University, Guangzhou, China; 3 Department of Blood Transfusion, Guangzhou Medical University, Guangzhou Women and Children’s Medical Center, Guangzhou, China; 4 Department of Pediatrics, The Affiliated Qingyuan Hospital (Qingyuan People’s Hospital), Guangzhou Medical University, Qingyuan, China; 5 Shanghai Public Health Clinical Center & Laboratory of RNA Epigenetics, Institutes of Biomedical Sciences & Department of General Surgery, Huashan Hospital, Cancer Metastasis Institute, Shanghai Medical College, Fudan University, Shanghai, China; 6 Department of Molecular Medical Center, Guangzhou Women and Children’s Medical Center, Guangzhou Medical University, Guangzhou, China; 7 Department of Traditional Chinese Medicine, Guangzhou Women and Children’s Medical Center, Guangzhou Medical University, Guangzhou, China; 8 Department of Gynecology, Guangzhou Women and Children’s Medical Center, Guangzhou Medical University, Guangzhou, China

**Keywords:** aspirin resistance, genetic susceptibility, inflammatory indices, kawasaki disease, platelet parameters

## Abstract

Kawasaki disease (KD) is the leading cause of acquired cardiovascular disease in children and is characterized by intense immune activation and platelet dysfunction. High platelet reactivity (HPR) is increasingly recognized as a biological basis of aspirin resistance (AR), which may increase the risk of adverse coronary outcomes, including coronary artery aneurysms (CAA). However, the hematologic dynamics and genetic determinants underlying AR in KD remain unclear. In this study, the association between AR and CAA was assessed using chi-square analysis. We compared platelet parameters between KD aspirin-resistant (KD-AR) and KD non-aspirin-resistant (KD-NAR) patients across different disease phases using linear mixed-effects models (LMM). Baseline complete blood count (CBC) derived inflammatory indices, including the systemic immune-inflammation index (SII), platelet-to-lymphocyte ratio (PLR), and neutrophil-to-lymphocyte ratio (NLR), were evaluated using restricted cubic spline (RCS) and receiver operating characteristic (ROC) analyses. Integrated transcriptomic and expression quantitative trait loci (eQTL) analyses were performed to identify candidate genetic factors associated with the KD-AR phenotype. The results showed that AR was significantly associated with CAA formation. LMM showed significant phase-dependent changes in platelet parameters, with distinct longitudinal trajectories between KD-AR and KD-NAR patients. Between-group differences were mainly observed during the subacute phase (D7–14), when KD-AR patients showed lower platelet count (PLT) and plateletcrit (PCT), but higher platelet distribution width (PDW) and platelet large cell ratio (PLCR). Baseline SII, PLR, and NLR were significantly elevated in KD-AR patients (all *P* < 0.001). RCS analyses demonstrated significant overall associations between these indices and AR risk (all *P*_overall_ < 0.001). ROC analyses showed moderate discrimination for SII (AUC = 0.702) and NLR (AUC = 0.722), whereas PLR showed lower performance (AUC = 0.626). *MBP* was consistently upregulated in HPR-associated samples, and eQTL integration identified *MBP/rs8090438* as a candidate variant linked to KD-AR. These findings suggest that AR in KD represents a multifactorial phenotype involving immune-driven platelet dysregulation and genetic susceptibility. Baseline inflammatory indices, particularly NLR and SII, may assist in early identification of KD patients at increased likelihood of AR.

## 1 Introduction

Kawasaki disease (KD), a systemic inflammatory disorder predominantly affecting children under 5 years of age, exhibits a significantly higher incidence in Asian populations (Rife and Gedalia, 2020). As the leading cause of acquired cardiovascular disease in childhood, KD may progress to coronary artery aneurysms (CAA) in approximately 25% of untreated cases (Fuller, 2019). Occlusive thrombosis secondary to CAA represents the primary contributor to KD mortality (Miura et al., 2018), closely associated with high platelet reactivity (HPR) (Shulman and Rowley, 2015). HPR is defined as a state in which platelets remain in relatively activated and prone to aggregation despite ongoing antiplatelet therapy. this condition reflects incomplete suppression of key platelet activation pathways (particularly adenosine diphosphate (ADP) or arachidonic acid (AA) signaling), which may cause clopidogrel resistance or aspirin resistance (AR) (Cattaneo, 2013). Patients with HPR exhibit persistently high platelet activation and aggregation potential, which is associated with an increased risk of ischemic complications, particularly in KD patients with CAA.

Aspirin, which possesses dual antiplatelet and anti-inflammatory properties, is indispensable for preventing thrombosis and vascular injury induced by KD. Although there is currently no evidence to support the use of moderate-to-high doses of aspirin during the acute febrile phase to mitigate the progression of CAA, the American Heart Association (AHA) recommended low-dose aspirin for a minimum of 6–8 weeks as a fundamental therapeutic approach to prevent coronary thrombosis and related major cardiovascular events (McCrindle et al., 2017). Currently, the efficacy of antithrombotic therapy for KD has been evaluated primarily through observational studies and small-scale trials (Assempoor et al., 2024; Li et al., 2021; Dallaire et al., 2017). Historically, the therapeutic benefits of intravenous immunoglobulin (IVIG) have been increasingly emphasized, often overshadowing the issue of AR (McCrindle et al., 2017). A Chinese clinical cohort study reported an incidence of AR in KD patients ranging from 8.05% to 9.75% (Meng et al., 2019). While few studies have directly quantified the relationship between AR and clinical adverse events, long-term follow-up data indicated that CAA resulting from childhood KD can persist into adulthood, leading to ischemic heart disease and acute coronary syndromes independent of traditional atherosclerotic mechanisms (Gordon et al., 2009). Consequently, identifying factors contributing to AR in KD patients undergoing long-term therapy is critical for mitigating cardiovascular complications.

Emerging evidence highlights the prognostic potential of platelet parameters in monitoring antiplatelet therapy efficacy (Freynhofer et al., 2015; Vinholt et al., 2014). Routinely measured platelet parameters, including platelet count (PLT), mean platelet volume (MPV), platelet distribution width (PDW), plateletcrit (PCT) and platelet large cell ratio (PLCR) are readily obtainable from complete blood counts (CBC). These parameters and their derivative biomarkers have been shown to reflect platelet activation status and therapeutic responsiveness to antiplatelet therapy (Vinholt et al., 2017; Monteiro et al., 2019; Lordkipanidzé et al., 2016; Zhou et al., 2024). Cost-effectiveness and widespread availability guarantee their clinical utility as indirect indicators of systemic inflammation, with established applications in cardiovascular diseases, thrombotic disorders, and chronic inflammatory conditions including infections and autoimmune diseases (Pasalic et al., 2016; López-Escobar et al., 2021; Chikovani et al., 2023). From the perspective of KD, recent studies have focused on leveraging inflammation-related hematological ratios, such as the neutrophil-to-lymphocyte ratio (NLR), platelet-to-lymphocyte ratio (PLR), the systemic immune-inflammation index (SII, platelet count × neutrophil count/lymphocyte count), to predict IVIG resistance in the acute stage (Liu and Wu, 2022; Yi et al., 2024). It has been reported that chronic inflammation (e.g., active atherosclerosis or immune-stimulated states) enhances platelet reactivity and promotes thromboxane A_2_ (TXA_2_) production via inflammatory cytokines, thereby reducing the clinical efficacy of aspirin (Zhao et al., 2021). Therefore, we aim to further investigate whether CBC-derived inflammatory biomarkers can serve as an early screening tool for AR in KD.

Simultaneously, accumulating genetic studies increasingly implicate hereditary factors as considerable determinants of AR (Zeng et al., 2024), and single nucleotide polymorphisms (SNPs) have been reported as critical contributors to this phenotype (Li et al., 2025; Liu et al., 2024; Ikonnikova et al., 2022; Gum et al., 2001). These genetic variants may impair drug bioavailability or target engagement, leading to suboptimal outcomes. Identifying SNPs through pharmacogenomic profiling could enable personalized dosing strategies, optimizing aspirin benefits in KD. Thus, exploring AR-related genetic markers in KD patients is also one of the objectives of this study.

These findings have spurred our exploration of KD in two interrelated domains: (1) the specific platelet parameters or other CBC-derived biomarker profiles associated with AR phenotype, and (2) the identification of genetic biomarkers correlated with AR.

## 2 Methods

### 2.1 Subjects

Based on the Women and Children’s Medical Center affiliated with Guangzhou Medical University from 2014 to 2021, our center accepted a total of 1,856 KD patients. All the subjects belonged to the Han ethnic group native to Southern China. The diagnostic criteria for KD were all following the guidelines released by the AHA in 2017 (McCrindle et al., 2017). Review medical records to make sure that all KD patients were treated with the standard regimen of aspirin (30–50 mg/kg/day) and a single intravenous dose of IVIG (2 g/kg) within the first 10 days from the beginning of fever.

**FIGURE 1:**
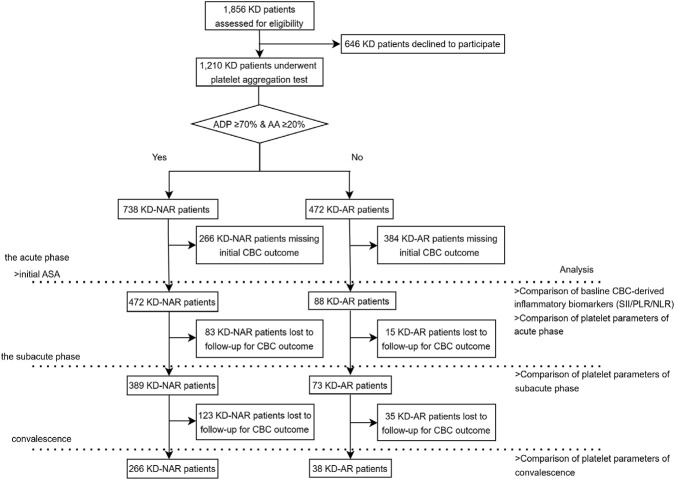
Study flowchart of participant inclusion and data availability. A substantial proportion of patients lacked available CBC data at baseline or during follow-up, resulting in varying sample sizes across different time points. Missing baseline CBC data primarily resulted from incomplete electronic medical records, whereas missing follow-up data was mainly due to follow-up at local hospitals or poor adherence to scheduled visits. Abbreviations: KD = Kawasaki disease, ADP = adenosine diphosphate, AA = arachidonic acid, ASA = aspirin, KD-AR = KD aspirin-resistant patients, KD-NAR = KD aspirin-non-resistant patients, CBC = complete blood counts, SII = the systemic immune-inflammation index, PLR = platelet-to-lymphocyte ratio, NLR = neutrophil-to-lymphocyte ratio.

During the study period, platelet aggregation test was standard practice at our institution to perform for all KD patients in the absence of specific contraindications. After excluding patients with follow-up visits and those with missing platelet aggregation test data, a total of 1,210 KD patients were included. The determination of AR in patients with KD should be based on the outcomes of platelet aggregation assays. AR was defined as a mean aggregation of ≥70% with 10 μM ADP and a mean aggregation of ≥20% with 0.5 mM AA (Gum et al., 2003). According to this criterion, a cohort of 1,210 KD patients was stratified into two groups: 738 KD aspirin-non-resistant (KD-NAR) patients and 472 KD aspirin-resistant (KD-AR) patients. We will analyze and compare platelet parameters in KD-AR and KD-NAR at different stages of KD, and further explore the manifestation of CBC-derived inflammatory biomarkers in AR at the acute phase of KD (Figure 1).

Furthermore, 1435 healthy controls (HCs) were included to calculate the association between *rs8090438* and KD susceptibility (Supplementary Table S1). HCs were randomly selected from children undergoing routine physical examinations and were matched with KD patients based on gender and age.

The study conformed to the Declaration of Helsinki and was performed with the approval of the Ethics Committee of the Women and Children’s Medical Center affiliated with Guangzhou Medical University (approval numbers: 2014073009, 2018052702, 2021093A01). Prior to the study, informed consent was acquired from the legal guardian of each participant.

### 2.2 Platelet aggregation

For KD patients, venous blood was drawn and placed in tubes (2–4 mL) with anticoagulant citrate dextrose solution (ACD), followed by treatment with 75 nM prostaglandin E1 (PGE1, Catalogue No: HY-B0131, MEC) to prevent platelet activation. Blood samples were collected 3 days after aspirin administration. The final aspirin dose was administered within 1–24 h prior to blood collection. Room-temperature blood samples were processed within 1 h of collection.

### 2.3 Platelet parameters examination

For each patient, 2 mL of venous blood was collected into collection tubes containing Dipotassium EDTA (1.5 mg/mL) for CBC and immature platelets testing. CBC was analyzed within 2 h of sampling by an automatic blood cell counter (Sysmex XE-5000, Sysmex, Kobe, Japan). The time points for monitoring CBC were as follows: before aspirin treatment (baseline), 7–14 days after aspirin treatment (the subacute phase of KD), and 28–56 days after aspirin treatment (the recovery phase of KD).

### 2.4 Data collection

The mRNA expression profiling microarray data were sourced from the Gene Expression Omnibus (GEO) database (http://www.ncbi.nih.gov/geo ). This database is publicly available and is exempt from ethical review. A search of the GEO database was conducted using the keywords “HPR” and “AR” to identify mRNA expression profiling microarray datasets pertinent to platelet reactivity. In accordance with the study objectives, this research incorporated two datasets, GSE66426 and GSE32226 (Figure 5A), for further analysis. GSE66426 includes transcriptomic profiles from aspirin-treated cardiovascular patients stratified by platelet reactivity, while GSE32226 comprises expression data from coronary artery disease patients receiving antiplatelet therapy with documented platelet response phenotypes. These datasets were selected as established human models of platelet hyperreactivity. Each dataset was utilized to identify differentially expressed genes, followed by an intersection analysis.

Patients' clinical information was acquired through a review of the medical record system.

### 2.5 Identification of differentially expressed genes (DEGs)

DEG analysis was conducted separately within each dataset using the GEO2R online tool based on the limma package. Given the use of different microarray platforms, expression matrices were not merged across datasets. DEG was identified with *P* < 0.05 of fold changes (FC) > 1.2 as the significance thresholds for initial exploratory screening. Genes consistently differentially expressed in both datasets were retained for downstream analyses to enhance robustness and reduce platform-specific bias. The Database for Annotation, Visualization and Integrated Discovery (DAVID) was used to perform functional enrichment analyses, including analysis of Gene Ontology (GO) and Kyoto Encyclopedia of Genes and Genomes (KEGG). Related diagrams were performed by R package “ggplot2”.

### 2.6 Analysis of functional expression quantitative trait loci (eQTL)

205 eQTLs were downloaded from GTEx (https://www.gtexportal.org/home/). eQTLs was evaluated using VannoPortal (http://mulinlab.org/vportal) and QTLbase (http://www.mulinlab.org/qtlbase). The linkage disequilibrium (LD) blocks were calculated among the East Asian population (“EAS”), Ad Mixed American population (“AMR”), and European population (“EUR”), utilizing the R package “LDlinkR”.

### 2.7 Extracting DNA and genotyping polymorphism

Genomic DNA was extracted from peripheral blood mononuclear cells (PBMCs) using the TIANamp Blood DNA Kit (Tiangen Biotech, Beijing, China). DNA concentration and quality were assessed prior to genotyping.

Genotyping of *rs8090438* was performed using the Sequenom MassARRAY iPLEX Gold system (Agena Bioscience, San Diego, CA, USA). In addition, quantitative polymerase chain reaction (qPCR) was used solely for DNA quality assessment. qPCR reactions were conducted in 384-well plates using the ABI QuantStudio 6 Real-Time PCR System (Thermo Fisher Scientific, USA).

The PCR conditions consisted of an initial denaturation at 95 °C for 5 min, followed by 45 cycles of denaturation at 95 °C for 15 s and annealing/extension at 60 °C for 1 min.

### 2.8 Statistical analysis

Statistical analyses were performed using SPSS version 26.0 (IBM Corp., Armonk, NY, USA), R software version 4.5.1 (R Foundation for Statistical Computing, Vienna, Austria), GraphPad Prism version 9.5 (GraphPad Software, San Diego, CA, USA), PLINK version 1.9 (Massachusetts General Hospital, Boston, MA, USA), and SAS version 9.4 (SAS Institute Inc., Cary, NC, USA).

Continuous variables were expressed as mean ± standard deviation (SD) for normally distributed data and as median with interquartile range (IQR) for skewed distributions. Categorical variables were presented as frequencies or percentages. Normality of continuous variables was assessed using the Shapiro-Wilk test. Between-group comparisons were conducted using the two-tailed Student’s t test for normally distributed variables and the Mann-Whitney U test for non-normally distributed variables.

For patients with multiple transthoracic echocardiography (TTE) examinations, the record documenting the most severe CAA findings was included for statistical analysis. The chi-square test was used to calculate the association between CAA and AR. Missing data were primarily observed in baseline CBC variables and follow-up platelet measurements (Figure 1). Baseline characteristics were compared between patients with and without available CBC data to assess potential selection bias.

Longitudinal changes of five platelet parameters (PLT, PDW, MPV, PCT, and PLCR) across three disease phases (D0, day before aspirin treatment, the acute phase; D7-14, 7–14 days after aspirin administration, the subacute phase; D28-56, 28–56 days after aspirin administration, the convalescent phase) were analyzed using linear mixed-effects models (LMM) by SPSS. Time and group (KD-NAR vs. KD-AR) were specified as fixed effects, and subject was included as a random effect to account for within-subject correlation. An unstructured covariance matrix was applied for repeated measures, and parameters were estimated using restricted maximum likelihood (REML). LMMs are generally considered robust to incomplete repeated measurements under the missing at random (MAR) assumption, thereby allowing inclusion of all available data without formal imputation. Post hoc simple effects were examined using Bonferroni-adjusted pairwise comparisons of estimated marginal means (EMMs) within each phase. The primary inference was based on the time × group interaction in the LMM.

Restricted cubic spline (RCS) analyses with four knots were performed to explore potential nonlinear associations of SII, PLR, and NLR with the risk of AR by R software. Sensitivity analyses using alternative numbers of knots (knot = 3/4/5) yielded consistent effect patterns, supporting the robustness of the spline models. Receiver operating characteristic (ROC) curve analyses were performed using SPSS to assess the discriminative performance of each CBC-derived inflammatory biomarker for distinguishing KD-AR from KD-NAR patients. The area under the ROC curve (AUC) and corresponding 95% confidence intervals (CIs) were calculated. AUC estimates were derived from univariable logistic regression models.

Genetic association analyses were conducted using PLINK and SAS. Hardy-Weinberg equilibrium (HWE) was assessed, with *P* > 0.05 indicating acceptable genotyping quality. Associations between the *rs8090438* polymorphism and AR, as well as susceptibility to KD, were evaluated under multiple genetic models, including the allelic model (ALLELIC), the 2df genotypic model (GENO), and the co-dominant model (CODOM), additive model (ADD), dominant model (DOM) and recessive model (REC). A two-sided *P* < 0.05 was considered statistically significant. False discovery rate (FDR) correction was applied to adjust the *P* values for multiple testing, with *P*_FDR_ < 0.01 considered statistically significant.

## 3 Results

### 3.1 General characteristics

**FIGURE 2:**
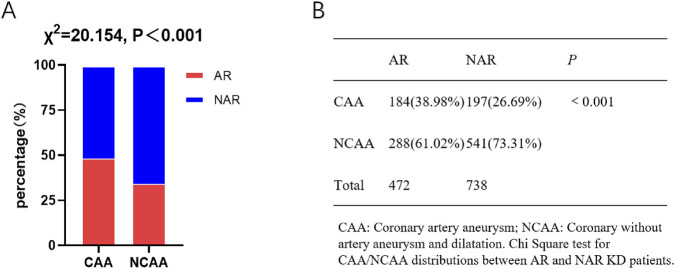
Correlation analysis between CAA and AR. **(A)** Stacked bar chart comparing the percentages of AR and NAR patients between the CAA and NCAA groups. **(B)** Table summarizing the corresponding patient counts, percentages, and P value. The chi-square test showed a significant difference in AR/NAR distribution between the two groups, suggesting that AR is significantly associated with CAA. Abbreviations: KD = Kawasaki disease; AR = aspirin resistance; NAR = non-aspirin resistance; CAA = coronary artery aneurysm; NCAA = no coronary artery aneurysm or dilation.

General information of KD patients was collected including the age of the disorder onset, gender and coronary artery outcomes from TTE during visits (Table 1). Coronary artery outcomes of KD patients were categorized according to their Z score: no coronary artery damage (Z < 2), coronary artery dilation (2 < Z < 2.5), small CAA (2.5 ≤ Z < 5), medium CAA (5 ≤ Z < 10, and absolute dimension <8 mm), and giant CAA (Z ≥ 10, or absolute dimension ≥8 mm) (McCrindle et al., 2017). Table 1 indicated that the KD-AR and KD-NAR cohorts exhibited pathogenics characteristics consistent with those of general KD patients, notably a higher prevalence among males under 5 years of age. The incidence of CAA was relatively consistent with that reported in external general KD cohorts (Haiyan et al., 2022). The chi-square test results indicated that the AR was significantly associated with the development of CAA (*P* < 0.05) (Figure 2).

**TABLE 1:** General Characteristics distribution in KD-AR and KD-NAR.

Characteristic	KD-NAR (n = 738)		KD-AR (n = 472)	
	(n)	(%)	(n)	(%)
Total	738	100.00	472	100.00
Gender				
Male	497	67.34	301	63.77
Female	241	32.66	171	36.23
Age				
mean ± SD	24.00 ± 20.27		26.87 ± 21.03	
≤5 years-old	708	95.93	451	95.55
>5 years-old	30	4.07	21	4.45
Coronary artery outcomes				
Giant aneurysm	25	3.39	23	4.87
Medium aneurysm	55	7.45	24	5.08
Small aneurysm	50	6.78	66	13.98
Transient dilation	67	9.08	71	15.04
No dilation	541	73.31	288	61.02

Abbreviations: KD = Kawasaki disease, KD-AR = KD aspirin-resistant patients, KD-NAR = KD aspirin-non-resistant patients, SD = standard deviation.

### 3.2 Longitudinal changes in platelet parameters between KD-NAR and KD-AR groups based on linear mixed-effects models (LMM)

Linear mixed-effects models (LMM) were used to assess longitudinal changes in platelet parameters (PLT, MPV, PDW, PCT, and PLCR) across three disease phases (D0, D7-14, and D28-56) between the KD-NAR and KD-AR groups. No significant differences were observed in baseline characteristics in terms of sex distribution, age, or coronary artery outcomes between patients with and without available baseline CBC data (all *P* > 0.05) (Supplementary Table S11). All available repeated measurements were included in the analyses.

#### 3.2.1 Overall fixed effects

The LMM demonstrated significant main effects of phase for all five platelet parameters, including PLT (F = 41.287, *P* < 0.001), MPV (F = 3.098, *P* = 0.047), PDW (F = 25.002, *P* < 0.001), PCT (F = 29.360, *P* < 0.001), and PLCR (F = 27.808, *P* < 0.001). A significant main effect of aspirin responsiveness was observed for PLT (F = 13.136, *P* < 0.001) and PCT (F = 12.621, *P* < 0.001), whereas no significant group effect was detected for MPV, PDW, or PLCR.

**FIGURE 3:**
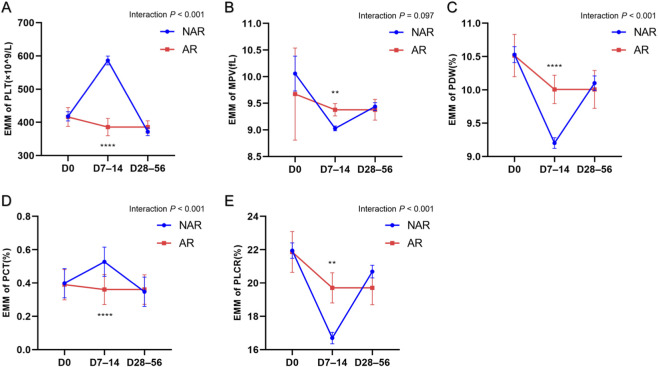
Longitudinal trajectories of platelet parameters estimated by LMM. **(A–E)** LMM-adjusted longitudinal trajectories corresponded to PLT, MPV, PDW, PLCR, and PCT, respectively. Data are presented as EMMs ±SE for the NAR and AR groups across three disease phases (D0, D7-14, and D28-56). Interaction *P* values shown in the upper right corner of each figure represented the phase × group interaction effects derived from the LMM. Asterisks indicated Bonferroni-adjusted simple effects comparing AR and NAR groups within each phase (^**^ *P* < 0.01, ^****^ *P* < 0.0001). Abbreviations: LMM = linear mixed-effects models, PLT = platelet count, PCT = plateletcrit, PDW = platelet distribution width, PLCR = platelet large cell ratio, MPV = mean platelet volume, KD-AR = KD aspirin-resistant patients, KD-NAR = KD aspirin-non-resistant patients, EMMs = estimated marginal means, SE = standard error.

Notably, significant phase × group interactions were identified for PLT (F = 38.829, *P* < 0.001), PDW (F = 8.275, *P* < 0.001), PCT (F = 29.839, *P* < 0.001), and PLCR (F = 10.269, *P* < 0.001). In contrast, no significant interaction effect was observed for MPV (F = 2.352, *P* = 0.097) (Figures 3A–E; Supplementary Table S12A).

#### 3.2.2 Bonferroni-adjusted simple effects

Bonferroni-adjusted pairwise comparisons demonstrated that between-group differences were primarily observed during the subacute phase (D7-14) (Figures 3A–E; Supplementary Table S12C). At this stage, KD-AR patients showed lower PLT (β = −200.613, 95% CI: -251.111 to −150.114, *P* < 0.001) and lower PCT (β = −0.167, 95% CI: -0.211 to −0.122, *P* < 0.001), but higher PDW (β = 0.805, 95% CI: 0.357 to 1.253, *P* < 0.001) and higher PLCR (β = 3.005, 95% CI: 1.096 to 4.914, *P* = 0.002). No significant between-group differences were observed at D0 or D28–56 (all *P* > 0.05).

Although the interaction for MPV was not statistically significant (F = 2.352, *P* = 0.097), a modest increase in MPV was observed in KD-AR patients at D7–14 (β = 0.350, 95% CI: 0.117 to 0.582, *P* = 0.003).

### 3.3 Association and discriminative performance of SII, PLR and NLR for early identification of AR in KD patients

In this study, we compared three CBC-derived biomarkers, including SII, PLR and NLR among AR and NAR groups in patients with KD inflammatory condition to further explore whether these indexes have potential to early identify AR phenotype.

**FIGURE 4:**
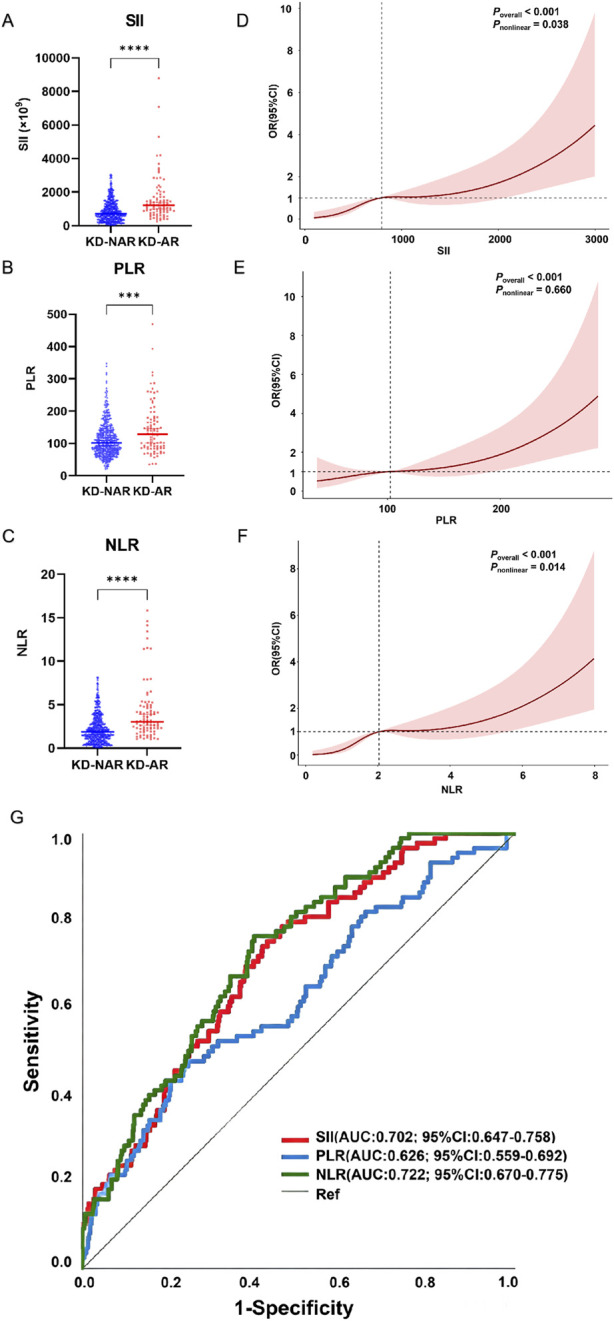
Baseline inflammatory biomarkers and their association with AR in KD. **(A–C)** Comparison of baseline SII, PLR, and NLR between KD-AR and KD-NAR. The data are presented as the median with IQR. Statistical comparisons were performed using the Mann-Whitney U test (^***^ *P* < 0.001, ^****^ *P* < 0.0001). **(D–F)** RCS analyses (df = 3) illustrating the exposure–response relationships between baseline SII **(D)**, PLR **(E)**, and NLR **(F)** and the risk of AR. Solid lines represent adjusted OR, and shaded areas indicate 95% CI. *P*_overall_ denotes the overall association, and *P*_nonlinear_ represents the test for non-linearity. **(G)** ROC curves evaluating the discriminative performance of SII, PLR, and NLR for distinguishing KD-AR from KD-NAR patients. SII (AUC = 0.702, 95% CI: 0.647–0.758), NLR (AUC = 0.722, 95% CI: 0.670–0.775), and PLR (AUC = 0.626, 95% CI: 0.559–0.692). The diagonal line represents the reference line (AUC = 0.5). Abbreviations: SII = the systemic immune-inflammation index, PLR = platelet-to-lymphocyte ratio, NLR = neutrophil-to-lymphocyte ratio, KD-AR = KD aspirin-resistant patients, KD-NAR = KD aspirin-non-resistant patients, IQR = interquartile range, RCS = Restricted cubic spline analyses, ROC = Receiver operating characteristic, AUC = area under curve, df = degrees of freedom, OR = odds ratio, CI = confidence interval.

SII, PLR and NLR were calculated prior to the initial aspirin treatment. All three indices were significantly higher in the KD-AR group compared with the KD-NAR group (all *P* < 0.001) (Table 2; Figures 4A–C). Restricted cubic spline (RCS) models with four knots (df = 3) demonstrated significant overall associations between SII, PLR, NLR and AR risk (all *P*_overall_ < 0.001) (Figures 4D–F). PLR exhibited a linear correlation with AR (*P*_nonlinear_ = 0.660) (Figure 4E). Both SII and NLR demonstrated evidence of a non-linear association (*P*_nonlinear_ < 0.05) (Figures 4D,F). Sensitivity analyses using alternative knot specifications (knot = 3/4/5) yielded consistent overall associations, and the general shape of the exposure-response curves remained stable (Supplementary Figure S1), indicating robustness of the findings.

**TABLE 2:** Comparison of the NLR, PLR and SII between KD-AR and KD-NAR groups before aspirin treatment.

Index	KD-NAR (n = 472)	KD-AR (n = 88)	*P*
SII	711(401–1221)	1222(817–1722)	**<0.0001**
PLR	101.72(73.65–137.13)	128.42(86.20–179.46)	**<0.001**
NLR	1.87(1.05–3.03)	3.05(2.20–4.83)	**<0.0001**

The data are presented as the median with IQR., Abbreviation; SII = the systemic immune-inflammation index, PLR = platelet-to-lymphocyte ratio, NLR = neutrophil-to-lymphocyte ratio, KD-AR = KD aspirin-resistant patients, KD-NAR = KD aspirin-non-resistant patients, IQR = interquartile range. Bolded text indicates a significant P value.

To assess discriminative performance, receiver operating characteristic (ROC) curve analyses were conducted. SII and NLR demonstrated moderate ability to distinguish KD-AR from KD-NAR patients (SII: AUC = 0.702, 95% CI: 0.647–0.758, *P* < 0.001; NLR: AUC = 0.722, 95% CI: 0.670–0.775, *P* < 0.001) (Figure 4G; Supplementary Table S13). In contrast, PLR exhibited lower discriminative performance (AUC = 0.626, 95% CI: 0.559–0.692, *P* < 0.001). Among the three indices, NLR showed the highest discriminative capacity.

Collectively, these findings indicate that elevated baseline SII and NLR levels are associated with increased AR risk and demonstrate moderate discriminative ability.

### 3.4 The process of DEG identification

**FIGURE 5:**
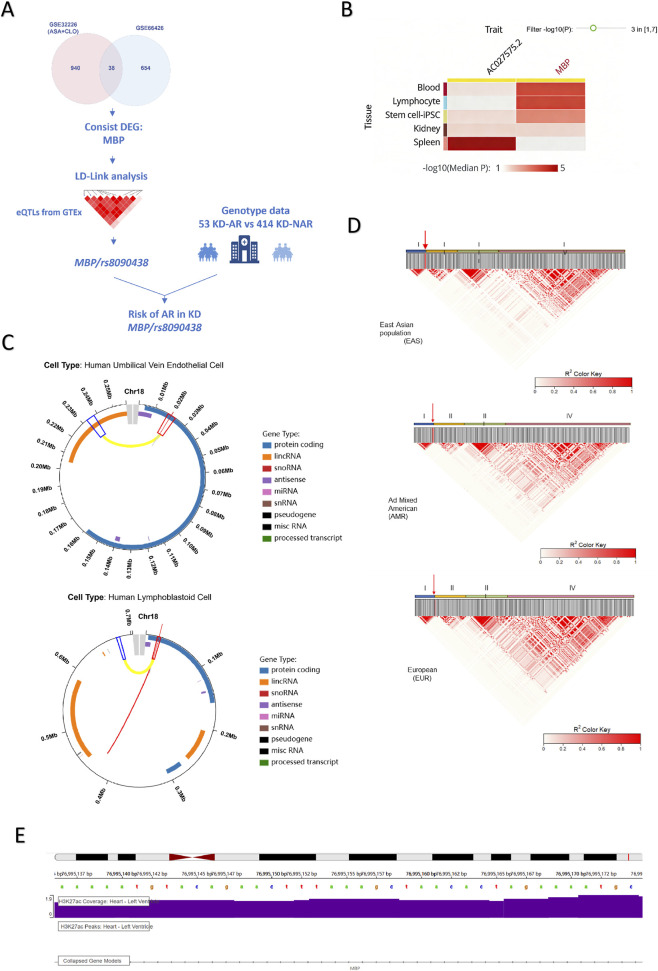
The process of determining *MBP/rs8090438* as an AR marker. **(A)** Identification of the potential association between *MBP/rs8090438* and AR in KD patients. We developed a comprehensive analytical framework. Initially, we identified two publicly available datasets related to AR and observed that *MBP* exhibited a notably significant fold change in individuals with HPR. Subsequently, we integrated the genomic location and pathogenic predictions of *MBP*, incorporating 205 expression eQTLs of *MBP*, along with their functional annotations. Furthermore, our analysis concentrated on the *MBP/rs8090438*, utilizing data from 51 KD-AR patients and 401 KD-NAR patients to explore its potential risk to AR in KD patients. **(B)** Tissue-specific distribution of rs8090438 according to the Qtlbase. This panel was redrawn by the authors based on QTLbase query results. **(C)** According to the Vannoportal, the panel displayed significant Hi-C interactions anchored at variant locus and associated epigenomic signal (from 127 Roadmap Epigenomics) in this locus from KD-related cells/tissues: human umbilical vein endothelial cell and human lymphoblastoid cell. This panel was redrawn by the authors based on VannoPortal annotation results. **(D)** Heatmaps of LD for “EAS”, “AMR”, and “EUR” populations related to the 205 total eQTLs of *MBP*, organized by genomic position. Detailed r2 values can be found in Supplementary Table S7. The red arrow pointed to the genomic position of *rs8090438*. **(E)** According to the GTEx, *rs8090438* (chr18:76,995,156(Grch38)) was located in the place with H3K27ac signal in heart tissue, which was regarded as an enhancer marker, suggesting *rs8090438* as a regulatory element in *MBP* gene. This panel was generated from the GTEx and the authors acknowledged the GTEx as the data and visualization source. Abbreviations: AR = aspirin resistance, HPR = high platelet reactivity, eQTL = the expression quantitative trait loci, LD = linkage disequilibrium, Hi-C = High-throughput chromosome conformation capture, KD-AR = KD aspirin-resistant patients, KD-NAR = KD aspirin-non-resistant patients, “EAS” = the East Asian population, “AMR” = Ad Mixed American population, “EUR” = European population.

The overall workflow of DEG identification was summarized in Figure 5A. After systematic screening of the GEO database, datasets GSE66426 and GSE32226 related to AR were included for subsequent analysis (Figure 5A; Supplementary Table S2). Differential expression analysis identified multiple DEGs in each dataset (Supplementary Tables S3-S4). Intersection analysis of the two DEG sets yielded 38 shared genes, among which 26 genes showed concordant directions of regulation (either upregulated or downregulated) in both datasets (Supplementary Table S5). Functional enrichment analysis of these 26 consistently regulated genes revealed significant enrichment in pathways related to the mitogen-activated protein kinase (MAPK) cascade, intracellular immune responses, and oxidative stress-related processes (Supplementary Table S6).

### 3.5 Pathogenic potential of *MBP/rs8090438* in cohort with HPR

Among the 26 genes showing concordant differential expression across both datasets, *MBP* was identified as a consistently upregulated gene (Supplementary Table S5). To further explore genetic variants potentially regulating *MBP* expression, eQTLs associated with *MBP* were retrieved from GTEx, yielding a total of 205 variants (Supplementary Table S7). Linkage disequilibrium (LD) analysis identified four major LD blocks in the “EAS” population (Figure 5D; Supplementary Table S8). To facilitate downstream annotation, one representative SNP from each LD block was selected based on LD independence (Supplementary Table S9). The variant *rs8090438* was located within block I and exhibited consistent LD patterns across three populations.

Functional annotation using VannoPortal and QTLbase indicated that *rs8090438* displayed high evolutionary conservation, supported by mamPhyloP (Phred Score 11.9902) and verPhyloP (Phred Score 11.6526). In addition, regulatory potential was suggested by Eigen_PC (Score 0.3813, Phred Score 12.4600) and fitCons (Score 0.0537, Phred Score 1.8046). The distribution of *rs8090438* was detected in platelet-derived tissue, including blood cells and pluripotent stem cells (Figure 5B). Vannoportal noted that *rs8090438* T allele potentially increased the binding affinity of several proteins including NR4A1 and RXRA, which were involved in immune and metabolic regulation, altering responses to environmental stimuli or metabolic states (Supplementary Table S10).

Epigenomic annotation showed that *rs8090438* was located in open chromatin regions with H3K27ac enrichment in heart tissue (Figure 5E). Chromatin interaction analysis based on Hi-C data from endothelial and lymphoblastoid cells revealed potential long-range interactions between *rs8090438* and distal genomic regions (Figure 5C).

### 3.6 Stratification analysis of *MBP/rs8090438* about KD susceptibility and KD-AR correlation

Stratification analysis was first performed to evaluate the association between *MBP/rs8090438* and KD susceptibility. No significant differences in genotype distribution were observed between KD patients and controls (Supplementary Table S1).

The association between *MBP/rs8090438* and AR was further evaluated in 51 KD-AR patients and 401 KD-NAR patients. Genetic association analyses were conducted under multiple inheritance models, including the allelic model (ALLELIC), 2df genotypic model (GENO), co-dominant model (CODOM), additive model (ADD), dominant model (DOM) and recessive model (REC). Significant associations between *MBP/rs8090438* and AR were observed across several genetic models, with the T allele conferring an increased risk of AR (Table 3). Notably, these associations remained statistically significant after FDR correction (*P*_FDR_ < 0.01), indicating robustness against multiple testing.

**TABLE 3:** Association between *MBP/rs8090438* and AR in KD patients.

SNP	Gene	A1/A2	Freq_A1	Freq_A2	HWE	Model	Genotype	AFF	UNAFF	OR	*P*_OR_	*P*_FDR_
***rs8090438***	*MBP*	T/A	0.25	0.36	0.807	ALLELIC	T/A	37/65	198/604	1.74(1.12–2.68)	**0.012**	0.015
						GENO					**0.0043**	**0.0072**
						CODOM	AA	17	229	Ref	-	
							TA	31	146	2.86(1.53–5.35)	**0.001**	**0.005**
							TT	3	26	1.55(0.43–5.66)	0.5	0.5
						ADD	TT/TA/AA	3/31/17	26/146/229	1.76(1.13–2.75)	**0.012**	0.015
						DOM	(TT + TA)/AA	34/17	172/229	2.66(1.44–4.92)	**0.0018**	**0.0045**
						REC	TT/(TT+TA)	3/48	26/375	0.90(0.26–3.09)	0.87	0.015

Abbreviations: A1/A2, risk/protective allele; Freq_A1/ Freq_A2, minor allele frequency of the SNP in KD-AR patients and KD-NAR patients, respectively; HWE, P value of Hardy–Weinberg equilibrium test; Model, ALLELIC, GENO, CODOM, DOM, ADD, and REC corresponds to the allelic model, 2df genotypic model, co-dominant model, additive model, dominant model and recessive model, respectively; AFF, allele count in KD-AR patients under different models; UNAFF, allele count in KD-NAR patients under different models; OR: odds ratio of the risk allele under different tests; CI, confidence interval; *P*_OR_ and *P*_FDR_, unadjusted *P* values under different tests and multiple testing correction (Bold text indicates *P*_OR_ < 0.05, *P*_FDR_ < 0.01).

## 4 Discussion

In accordance with the 2017 AHA Guideline, high-dose aspirin primarily exerts anti-inflammatory effects during the acute phase of KD, whereas low-dose aspirin is principally used for antiplatelet therapy in the subsequent stages (McCrindle et al., 2017). Accordingly, we systematically examined the clinical correlation between AR and platelet parameters, CBC-derived inflammatory markers, and genetic susceptibility in KD patients.

In this study, AR was defined as a mean aggregation of ≥70% with 10 μM ADP and ≥20% with 0.5 mM AA (Gum et al., 2003). This combined criterion differs from the traditional AA-only assessment, as ADP-induced platelet activation is predominantly mediated via P2Y1 and P2Y12 pathways, but can also promote AA release and secondary COX-1–dependent TXA_2_ generation, indicating a functional interaction between ADP signaling and the COX-1/TXA_2_ pathway (Jin et al., 2002). By incorporating both ADP and AA stimuli, this definition may better capture residual platelet reactivity and the clinically observable HPR phenotype, thereby reducing potential false-negative classification associated with single-pathway testing. This criterion has been widely applied in numerous studies to identify HPR high-risk patients. Given the lack of validated pediatric-specific criteria for AR in KD, and the similarity in platelet physiology and the mechanism of action of aspirin across age groups, we adopted these established adult criteria as a reference standard for evaluating AR in our KD cohort.

We identified a significant association between AR and the development of CAA. From a pathophysiological perspective, AR may reflect heightened platelet activation, increased inflammatory burden, or other circulatory disturbances (Davì and Patrono, 2007). In the context of vascular injury, alternative pathways, such as cyclooxygenase-2 (COX-2), may partly contribute to sustained platelet aggregation and thrombotic tendency even when COX-1-mediated TXA_2_ synthesis is adequately inhibited by aspirin (Hanjis et al., 2006). Similar mechanisms have been described in cardiovascular research, supporting the view of AR as a risk indicator for coronary artery disease rather than solely a measure of drug responsiveness (Patrono et al., 2005).

The longitudinal analysis revealed significant temporal variation in all platelet parameters across the disease course, with distinct phase × group interactions for PLT, PDW, PCT, and PLCR, indicating different dynamic trajectories between the AR and NAR groups. Notably, these differences were predominantly observed during the subacute phase (D7-14), when the AR group showed lower PLT and PCT levels but higher PDW and PLCR. Previous studies have shown that the subacute phase of KD is a critical stage characterized by persistent vascular inflammation and an elevated risk of coronary artery involvement (McCrindle et al., 2017; Rowley, 2011). In this context, the subacute phase has also been associated with active endothelial injury and enhanced platelet activation (Yu et al., 2022; Wang et al., 2025). The reduced PLT and PCT observed in AR patients may therefore be associated with increased platelet consumption under sustained inflammatory conditions, whereas elevated PDW and PLCR suggest greater platelet size heterogeneity and a higher proportion of larger, more reactive platelets with increased prothrombotic potential (Ligi et al., 2023; Pedersen et al., 2024). The absence of significant MPV differences suggests that distribution-based parameters may reflect platelet activation status more sensitively than MPV under inflammatory conditions (Gasparyan et al., 2011). Collectively, these findings suggest that AR in KD is associated with phase-specific alterations in platelet dynamics and heterogeneity during the subacute stage, highlighting this period as a clinically relevant window for monitoring platelet-related hematological changes.

SII, PLR and NLR were assessed prior to the initiation of aspirin therapy, thereby minimizing potential confounding effects of treatment on inflammatory status. These CBC-derived inflammatory markers were significantly elevated in KD-AR group, indicating that a heightened inflammatory burden precedes and may underlie impaired aspirin responsiveness. As composite indices integrating neutrophil activation, lymphocyte suppression, and platelet involvement, SII, PLR, and NLR have been widely used to reflect systemic inflammatory status in KD and other cardiovascular inflammatory conditions (Yontar and Mutlu, 2024; Lim et al., 2025). In the context of stable sensitivity analyses, RCS analysis demonstrated a statistically significant overall association between SII/PLR/NLR levels and AR risk (*P*_overall_ < 0.001), revealing a progressive increase in AR risk observed at higher marker levels. The results collectively demonstrate that CBC-derived inflammatory biomarkers SII/PLR/NLR show early elevation in KD-AR patients, indicating that vigilance should be exercised regarding the risk of AR induced by severe inflammation in KD. Furthermore, ROC curve analyses revealed that SII and NLR demonstrated moderate discriminative performance for distinguishing KD-AR from KD-NAR patients (AUC: 0.702 and 0.722, respectively), whereas PLR showed comparatively lower discriminatory capacity (AUC: 0.626). Although the discriminative ability was modest, these findings suggest that baseline inflammatory indices—particularly NLR and SII—may assist in the early identification of KD patients at higher likelihood of AR. Overall, our results support a close association between enhanced systemic inflammation and AR in KD, highlighting the potential value of routine CBC-derived inflammatory biomarkers as accessible adjunctive indicators for clinical risk stratification.

Beyond clinical parameters, we further explored genetic markers associated with the AR in the KD population. Previous studies have suggested that MBP is involved in immune regulation and may be linked to platelet activation through MAPK1-related pathways (Samiei et al., 1993). In conjunction with our DEG and enrichment results, *MBP* may represent a potential biological link between altered immune transcriptomic profiles and abnormal platelet activation observed in KD-AR. Through integrated bioinformatics and genetic analyses, *MBP/rs8090438* was identified as a regulatory genetic variant associated with the AR phenotype in KD. Emerging evidence indicates that variants in immune-related genes may indirectly influence platelet activation and antiplatelet drug responsiveness through inflammation-dependent mechanisms (Goodman et al., 2008). Similar gene-drug interaction patterns have been reported in other cardiovascular and inflammatory diseases, highlighting the contribution of host genetic background to individual variability in aspirin responsiveness (Dandona, 2014).

Limitations of this study should be acknowledged. First, insufficient data on thrombotic events were captured to allow for meaningful statistical analysis in this cohort. Second, although RCS modeling enabled a flexible assessment of exposure-response relationships and ROC analysis demonstrated moderate discriminative performance, the observed AUC values indicated that these biomarkers alone are insufficient for definitive clinical prediction. Therefore, SII, PLR, and NLR should be interpreted as adjunctive screening indicators rather than standalone predictive tools. In addition, no external validation cohort was included, and prospective studies are required to confirm the stability and generalizability of these findings. Finally, the genetic associations identified in this study should be interpreted with caution and warrant replication in larger, independent populations as well as mechanistic investigations to clarify their biological relevance.

## 5 Conclusion

In conclusion, our research findings suggest that AR in KD is a multi-factorial phenomenon propelled by systemic inflammation, dynamic platelet depletion, and genetic susceptibility, and it is correlated with an elevated risk of CAA. Monitoring inflammatory markers and platelet parameters, especially during the sub-acute phase, may contribute to the early identification of patients at high risk of AR.

## Data Availability

The datasets presented in this study can be found in online repositories. The names of the repository/repositories and accession number(s) can be found in the article/Supplementary Material.
