# Supplementary figures and images for: Platelet-related hematologic markers and genetic associations of aspirin resistance in kawasaki disease
Source: Front Mol Biosci. 2026 Apr 1;13:1807254. doi: 10.3389/fmolb.2026.1807254 (PMC13080441; doi:10.3389/fmolb.2026.1807254)

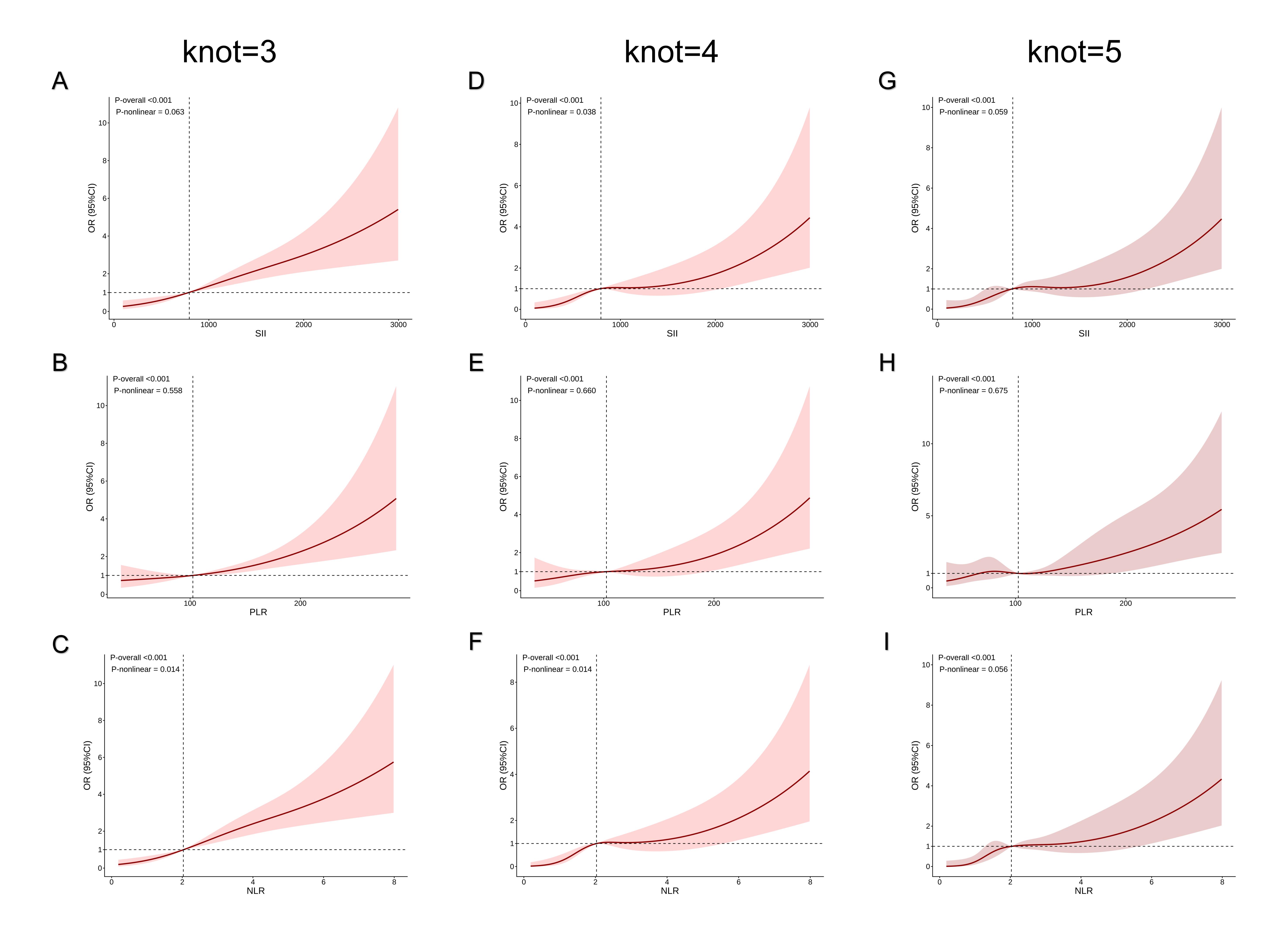

Supplement: Supplementary file 1 [file Image1.jpeg]
